# Diversity of endocervical microbiota associated with genital *Chlamydia trachomatis* infection and infertility among women visiting obstetrics and gynecology clinics in Malaysia

**DOI:** 10.1371/journal.pone.0224658

**Published:** 2019-11-18

**Authors:** Heng Choon Cheong, Polly Soo Xi Yap, Chun Wie Chong, Yi Ying Cheok, Chalystha Yie Qin Lee, Grace Min Yi Tan, Sofiah Sulaiman, Jamiyah Hassan, Negar Shafiei Sabet, Chung Yeng Looi, Rishein Gupta, Bernard Arulanandam, Sazaly AbuBakar, Cindy Shuan Ju Teh, Li Yen Chang, Won Fen Wong

**Affiliations:** 1 Department of Medical Microbiology, Faculty of Medicine, University of Malaya, Kuala Lumpur, Malaysia; 2 School of Pharmacy, Monash University Malaysia, Jalan Lagoon Selatan, Bandar Sunway, Selangor, Malaysia; 3 Department of Obstetrics and Gynecology, Faculty of medicine, University of Malaya, Kuala Lumpur, Malaysia; 4 Faculty of Medicine, SEGi University, Kota Damansara, Selangor, Malaysia; 5 School of Bioscience, Taylor’s University, Subang Jaya, Selangor, Malaysia; 6 Center of Excellence in Infection Genomics, South Texas Center for Emerging Infectious Diseases, University of Texas at San Antonio, San Antonio, Texas, United States of America; 7 Tropical Infectious Disease Research and Education Center, University of Malaya, Kuala Lumpur, Malaysia; GGD Amsterdam, NETHERLANDS

## Abstract

The cervical microbiota constitutes an important protective barrier against the invasion of pathogenic microorganisms. A disruption of microbiota within the cervical milieu has been suggested to be a driving factor of sexually transmitted infections. These include *Chlamydia trachomatis* which frequently causes serious reproductive sequelae such as infertility in women. In this study, we profiled the cervical microbial composition of a population of 70 reproductive-age Malaysian women; among which 40 (57.1%) were diagnosed with genital *C*. *trachomatis* infection, and 30 (42.8%) without *C*. *trachomatis* infection. Our findings showed a distinct compositional difference between the cervical microbiota of *C*. *trachomatis*-infected subjects and subjects without *C*. *trachomatis* infection. Specifically, significant elevations of mostly strict and facultative anaerobes such as *Streptococcus*, *Megasphaera*, *Prevotella*, and *Veillonella* in the cervical microbiota of *C*. *trachomatis*-positive women were detected. The results from the current study highlights an interaction of *C*. *trachomatis* with the environmental microbiome in the endocervical region.

## Introduction

Alongside *Trichomonas vaginalis*, *Neisseria gonorrhoeae*, and *Treponema pallidum*, genital *Chlamydia trachomatis* infection is one of the four most common curable sexually transmitted infections (STIs) in the world, with approximately 131 million cases of new infections being reported in year 2012 [1, 2]. The seroprevalence for *C*. *trachomatis* infection was high (45.5%) in Malaysia with a large percentage (94.4%) of infection among prostitutes [3]. Chlamydial infection also occurs frequently among women with fertility disorders and gynecological problems in Malaysia (22.7%-51.1%), indicating widespread infection within the country [4, 5].

Although a cure for *C*. *trachomatis* infection is achievable using appropriate antibiotics for most cases (≥ 97%) [6, 7], a large proportion of asymptomatic cases (50–70%) combined with high rates of reinfection remain the significant challenges to ongoing efforts targeted at preventing bacterial dissemination and reducing damages related to infection [8, 9]. *C*. *trachomatis* infections of the genital tract in females are characterized by a vast spectrum of genital tract pathologies that include mucopurulent cervicitis, urethritis, and salpingitis, which could further lead to pelvic inflammatory disease (PID), infertility, ectopic pregnancy and cervical cancer [10–12]. Further, genital chlamydial infection is also linked to preterm delivery and spontaneous abortion, as well as neonatal conjunctivitis [8, 13–15].

An increasing number of studies have highlighted disruption of vaginal microbiome as a predisposing factor for infection by urogenital pathogens. A healthy cervicovaginal microbiota is typically dominated by bacteria of the genus *Lactobacillus* [16]. *Lactobacillus* spp. exert their protective role in the female reproductive tract against the invasion of pathogenic microorganisms by maintaining the acidity of the mucosal environment, inhibiting the adhesion of pathogens, and producing bactericidal compounds such as hydrogen peroxide (H_2_O_2_) [17]. Under *in vitro* condition, *Lactobacillus* spp. are potent inhibitors of *C*. *trachomatis* largely due to their lactic acid producing capacities [18–20]. In case of bacterial vaginosis, a state of microbial imbalance within the vaginal environment where *Lactobacillus* spp. were replaced by other anaerobic bacteria has been linked to the increase transmission of STIs, including infections caused by *C*. *trachomatis*, *Neisseria gonorrhoeae*, *Trichomonas vaginalis*, human papillomavirus (HPV), and human immunodeficiency virus (HIV) [21–25].

Microbiota studies in recent years have shown that women with genital *C*. *trachomatis* infection exhibit increased colonization of anaerobic bacteria in the cervicovaginal region, although a causal relationship cannot be determined due to the use of a cross-sectional rather than a longitudinal study design. This suggests the presence of a crosstalk between microbiome community composition and the risk of chlamydial infection, which may dictate the subsequent course and outcome of female reproductive tract disorders [26–29]. However, most of the studies were conducted primarily among the European and South African populations, the data obtained are likely different from the microbiota diversity in the Asian cohort. In this present study, we evaluated the alteration of endocervical microbiome in association with *C*. *trachomatis* infection among a cohort of women in Malaysia by performing a 16S rRNA metagenomic sequencing analysis.

## Materials and methods

### Study population

Our patient cohort comprised 77 female subjects attending the gynecology outpatient clinics at the University of Malaya Medical Centre (a referral centre for subspecialties including early pregnancy, infertility, oncology and general gynaecology) from year 2010 to 2014. All patients were thoroughly briefed about the purpose of the study and written informed consents were obtained from each subject prior to participation in this study. The information pertinent to visits to gynecology clinic including reasons for referral, menstruation, symptoms of genital and urinary tract infection, obstetric and medical histories were documented prior to sample collection by physicians. Patients attending the gynecology clinics for different purposes, especially those diagnosed with infertility, were randomly sampled. From the cohort collection of 180 patients, those with low DNA concentration or insufficient DNA quantity were excluded. A total of 77 samples comprising of approximately 42 *C*. *trachomatis*-infected and 35 non-infected samples were then randomly selected from the total patient cohort. The criteria for inclusion were females at the reproductive age (18 to 40 years old), while the criteria for exclusion were positive urine pregnancy test, recent antibiotic therapy, and genital tuberculosis. The definition of primary infertility used in the present investigation is the inability to achieve conception after a minimum period of one year of regular sexual intercourse without the use of contraception, whereas secondary infertility refers to the failure to conceive after the last child birth. Ethical approval was granted by the Ethics Committee of the University of Malaya Medical Centre Medical Research Ethics Committee (MREC) before commencement of this project (Reference number 908.109).

### Specimen processing and diagnosis

Endocervical swabs were collected by the healthcare workers from the endocervix using UTM-RT universal transport media tubes (Copan, Brescia, Italy) and processed as described previously [5]. In brief, the transport tubes containing the endocervical swab was vortex mixed and the homogenate was centrifuged at 10,000× *g*. The resultant pellet was lysed and separated using phenol chloroform: isoamyl alcohol. DNA was precipitated with 1:10 volume of 3 M sodium acetate and isopropanol. After overnight storage at -20°C, the DNA pellet was washed and then eluted in 10 mM Tris-HCl, pH 8.5. Detection of genital *C*. *trachomatis* infection was performed using a combination technique of nested-PCR and RT-PCR as described previously [5] using primer pairs (Supporting Information S1 Table) specifically targeting the *C*. *trachomatis* MOMP and cryptic plasmid genes alongside a separate amplification of human β-globin gene that served as a positive control for successful DNA extraction. All tests were run along with a positive (bacterial DNA) and negative (non-template control) samples.

### 16S rRNA library preparation and HiSeq sequencing

16S rRNA library was prepared using the protocols outlined in the 16S metagenomic sequencing library preparation part 15044223-B (Illumina, San Diego, CA). Amplification of 16S rRNA V3-V4 hypervariable regions was conducted in a 25 μl PCR reaction containing 2.5 μl of template DNA (5 ng/μl), 5 μl of 1 μM V3-V4 forward primer (5ʹ-CCTACGGGNGGCWGCAG-3ʹ) and reverse primer (5ʹ-GACTACHVGGGTATCTA-3ʹ), and 12.5 μl of 2× HiFi hotstart readymix (KapaBiosystems, Wilmington, MA). The PCR cycling conditions consisted of an initial denaturation at 95°C for 3 min, 25 cycles of 95°C for 30 s, 55°C for 30 s, and 72°C for 30 s, followed by a final extension at 72°C for 5 min. PCR products were analyzed by 1.5% agarose gel electrophoresis and subsequently purified using Agencourt AMPure XP beads (Beckman Coulter, Brea, CA).

A second PCR was performed to introduce unique dual indices to the ends of the amplified DNA. The PCR master mix comprised 5 μl of purified PCR products, 5 μl of Nextera XT index primer 1 and 2 (Illumina), 25 μl of 2× HiFi hotstart readymix (KapaBiosystems), and 10 μl of sterile water to a final reaction volume of 50 μl. Samples were amplified using the following protocol: 95°C for 3 min, 8 cycles of 95°C for 30 s, 55°C for 30 s, and 72°C for 30 s, as well as a final extension at 72°C for 5 min. DNA libraries were re-purified with Agencourt AMPure XP beads (Beckman Coulter) and DNA quantity was measured with Qubit dsDNA HS assay (Life Technologies, Carlsbad, CA). Libraries were loaded on Agilent 2100 Bioanalyzer (Agilent Technologies, Palo, Alto, CA) to evaluate average fragment size and yield using Agilent high sensitivity DNA kit (Agilent Technologies) in accordance with the manufacturer’s protocol. Library qualities were assessed with an Mx3000P qPCR system (Agilent Technologies) using a library quantification kit (KapaBiosystems).

Following initial library quality control on the MiSeq system with the MiSeq V2 reagent kit (Illumina), the libraries were pooled at equimolar concentration with 15% PhiX spike-in. The final library was then subjected to paired-end 2× 250 bp sequencing on a HiSeq 2500 platform using HiSeq rapid SBS kit V2 (Illumina).

### Sequencing data analysis

Raw reads generated from Illumina paired-end sequencing were processed and quality filtered by using Mothur version 1.4.0 [30]. Briefly, 46,503,524 paired-end sequences were joined into contigs using Make.contigs command. Sequences were filtered by using the following criteria: minimum 20 bp overlapped, maximum 6 bp homopolymer and no ambiguous nucleotide. Approximately 50% of the sequences was removed based on the filter and a total of 23,029,897 sequences were used for subsequent procedures.

Clustering and assignment of operational taxonomic unit (OTUs) was performed using Recreated SILVA SEED Database Release 132 (https://mothur.org/wiki/Silva_reference_files). Chimeric sequences were identified and removed using VSEARCH within the Mothur pipeline. The final dataset consisted of 669 OTUs from 10141568 sequences (min = 40476; max = 211854), with mean length of 464 bp. Sequences for OTUs were subjected to a search in NCBI’s database using the BLASTn algorithm to identify taxa at the species level (http://blast.ncbi.nlm.nih.gov/Blast.cgi). For ease of comparison, the data was rarefied to equal depth of 40476 sequences per sample. Alpha diversity metrices including Shannon Diversity Index, Simpson Diversity Index and Pielou’s Evenness were calculated and projected in bar graphs using MicrobiomeSeq package. In addition, bar charts were constructed using phyloseq package [31] to display the proportional differences in genus and phylum across groups.

The beta-diversity (overall differences in bacterial composition across infection i.e. non-infected versus *C*. *trachomatis*-infected; and fertility i.e. fertile versus infertile/miscarriage status) was evaluated using Canonical analysis of principal coordinates (CAP) and Permutational Multivariate Analysis of Variance (PERMANOVA). Further, taxa showing significant differences in abundance between infection status i.e. chlamydial-infected and non-infected patients were identified using negative log binomial model implemented in DESeq2 R package [32]. Correction of multiple correction was conducted using Benjamini-Hochberg (BH) procedure implemented in DESeq2. Differentially expressed phylum genus and OTUs were selected by using BH adjusted P-value cut off of 0.01.

## Results

### Patients’ demographics

The study cohort consisted of 77 voluntarily participating women of reproductive age (20–44) presenting to the Obstetrics and Gynecology clinic at the University of Malaya Medical Center from the year 2010 to 2014. After quality filtering and chimera removal, 7 samples which generated fewer than 40,000 16S rRNA gene amplicon reads were omitted from further analyses. The final population comprised 70 subjects with ages ranging from 20 to 44 (mean age: 31.4; IQR: 27–35). Of the 70 subjects enrolled, 50 were married, 3 were divorced, and the remaining 17 women had not disclosed their marital status. The study cohort comprised 74.3% (52/70) subjects from the Malay ethnic group, Chinese and Indians each contributing 11.4% (8/70), while 2.4% (2/70) were of other ethnic backgrounds, which describes the demographic diversity of the multiethnic communities in Malaysia (Table 1). Among all, 51.4% (34/70) subjects were grouped as infertile including 20 diagnosed with primary or secondary infertility and 14 patients with miscarriage experience. Besides, 22.9% (16/70) of the subjects reported menstrual irregularity.

**Table 1:** Patient demographic characteristics. The demographics and clinical characteristics were analyzed using GraphPad PRISM software 7.0.

Parameters	All (*n* = 70)	No infection (*n* = 30)	*C*. *trachomatis* infection (*n* = 40)	*P-*value	OR (95% CI)
Age (years)					
Mean [IQR]	31.4 [27–35]	31.4 [27–36]	31.45 [28–35]	0.9689 ^*n*.*s*.^	
Maximum	44	44	42		
Minimum	20	20	21		
Marital status					
Married	50	19 (38%)	31 (62%)	0.2850 ^*n*.*s*.^	1.994 (0.6979–5.698)
Single	3	1 (33%)	2 (67%)	> 0.9999 ^*n*.*s*.^^.^	1.526 (0.1319–17.66)
Unknown	17	10 (59%)	7 (41%)	0.1628 ^*n*.*s*.^	0.4242 (0.1392–1.293)
Ethnicity					
Malay	52	23 (42%)	29 (58%)	0.7861 ^*n*.*s*.^^.^	0.8024 (0.2686–2.397)
Chinese	8	5 (69%)	3 (31%)	0.2748 ^*n*.*s*.^	0.4054 (0.08879–1.851)
Indian	8	2 (25%)	6 (75%)	0.4517 ^*n*.*s*.^	2.471 (0.462–13.21)
Others	2	0 (0%)	2 (100%)	0.5031 ^*n*.*s*.^	3.961 (0.1833–85.61)
Parameters					
Fertile	36	26 (72%)	10 (28%)		
Infertile	34	4 (12%)	30 (88%)	< 0.0001***	19.50 (5.459–69.66)
- 1° or 2°	20	0 (0%)	20 (100%)	< 0.0001***	103.5 (5.722–1871)
- Miscarriage	14	4 (29%)	10 (71%)	0.0089**	6.5 (1.652–25.58)
Menstrual cycle					
Regular	54	26 (48%)	28 (52%)		
Irregular	16	4 (25%)	12 (75%)	0.1507 ^*n*.*s*.^	2.7186 (0.7972–9.734)

Differences between categorical data were examined using Fisher’s exact test. P-value was computed using Student’s *t*-test, and statistical significance was established when P < 0.05*, P < 0.01** and P < 0.001***.

*n*.*s*.: non-significant. Odds ratio (OR) and 95% confidence interval (CI) were calculated.

### The relationship of *C*. *trachomatis* infection with infertility

A total of 40 out of 70 participants were infected with genital *C*. *trachomatis* based on the diagnostic test result. No significant correlation between chlamydial infection was detected with demographics such as age, marital status, as well as ethnicity (Table 1). When the subjects were grouped by infertility status, 88% (30/34) of the subjects from the infertile group was infected by *C*. *trachomatis*, as opposed to only 28% (10/36) in the fertile group. Indeed, a significant association between genital chlamydial infection and infertility was established (OR:19.50, 95% CI:5.459–69.66, *P* < 0.0001***). It is noteworthy that high prevalence of *C*. *trachomatis* infection was detected among the patients suffering from primary or secondary infertility (OR:103.50, 95% CI:5.722–1871, *P* < 0.0001***) and miscarriage (OR:6.50, 95% CI:1.652–25.58, *P* < 0.0089**), which were 100% (20/20) and 71% (10/14), respectively. However, the prevalence of *C*. *trachomatis* infection was not associated with menstrual health.

### Association between *C*. *trachomatis* infection and the endocervical microbial community composition

**Fig 1:**
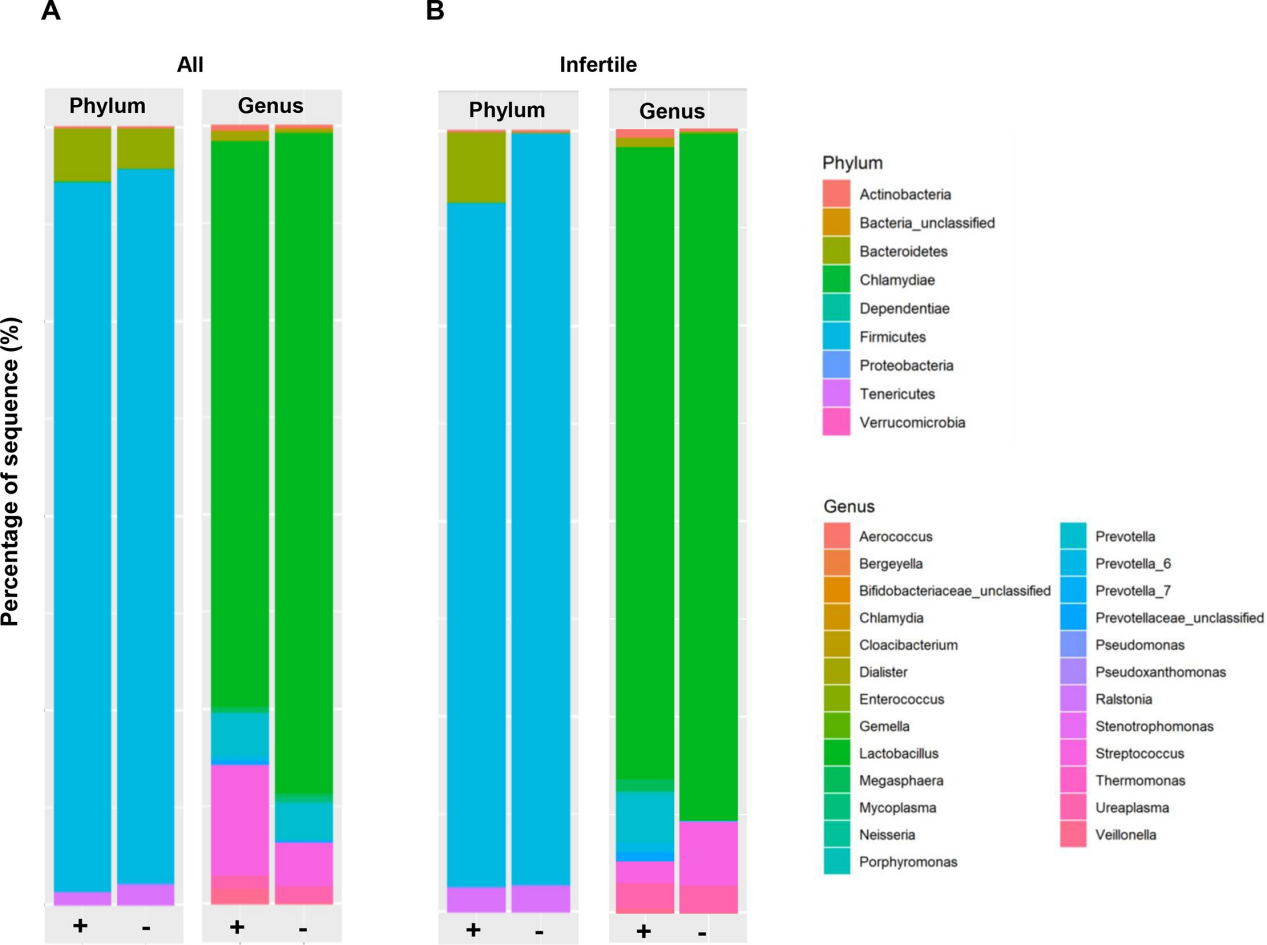
Taxonomical classification and diversity of cervical microbiome associated with *C*. *trachomatis* infection in females. (A) The overall phylum- or genus-based distributions of bacterial taxa in all subjects with (+) and without (-) genital *C*. *trachomatis* infection. (B) The phylum- or genus-based distributions of bacterial taxa among the infertile cohort, with (+) or without (-) genital *C*. *trachomatis* infection. Reads that were not aligned to reference alignment are denoted as “unclassified”.

The phylum and genus based taxonomic distributions are provided in Fig 1 and compared using negative log binomial model. Among all the patients, the dominant phyla were Firmicutes, followed by Bacteroidetes and Tenericutes (Fig 1A). When the comparison was made at phylum level, an enriched level of *Chlamydiae* was observed in the subjects diagnosed with (+) genital *C*. *trachomatis* infection; while those without (-) genital *C*. *trachomatis* infection was found to harbor higher levels of Tenericutes and Proteobacteria. At genus level, higher level of *Delftia*, *Streptococcus*, *Pseudomonas*, *Cloacibacterium*, *Prevotella*, *Veillonella*, *Megasphaera*, *Ureaplasma*, and *Ralstonia* were obvious among the subjects with *C*. *trachomatis* infection (+) compared to the group without chlamydial infection (Fig 1A).

When we restricted our analysis to infertile women, elevated level of phyla Bacteroidetes was detected the *C*. *trachomatis*-infected samples (+). A lower prevalence of genus *Megasphaera* was detected among the subjects with *C*. *trachomatis* infection (+) in comparison to the non-infected group (Fig 1B).

### Genital *C*. *trachomatis* infection is accompanied by increased endocervical colonization of *Lactobacillus* sp., *Streptococcus agalactiae*, *Prevotella colorans*, *Pseudomonas* sp., *Veillonella* sp., and *Delftia* sp.; and reduced *Aerococcus christensenii* and *Stenotrophomonas maltophilia*

**Fig 2:**
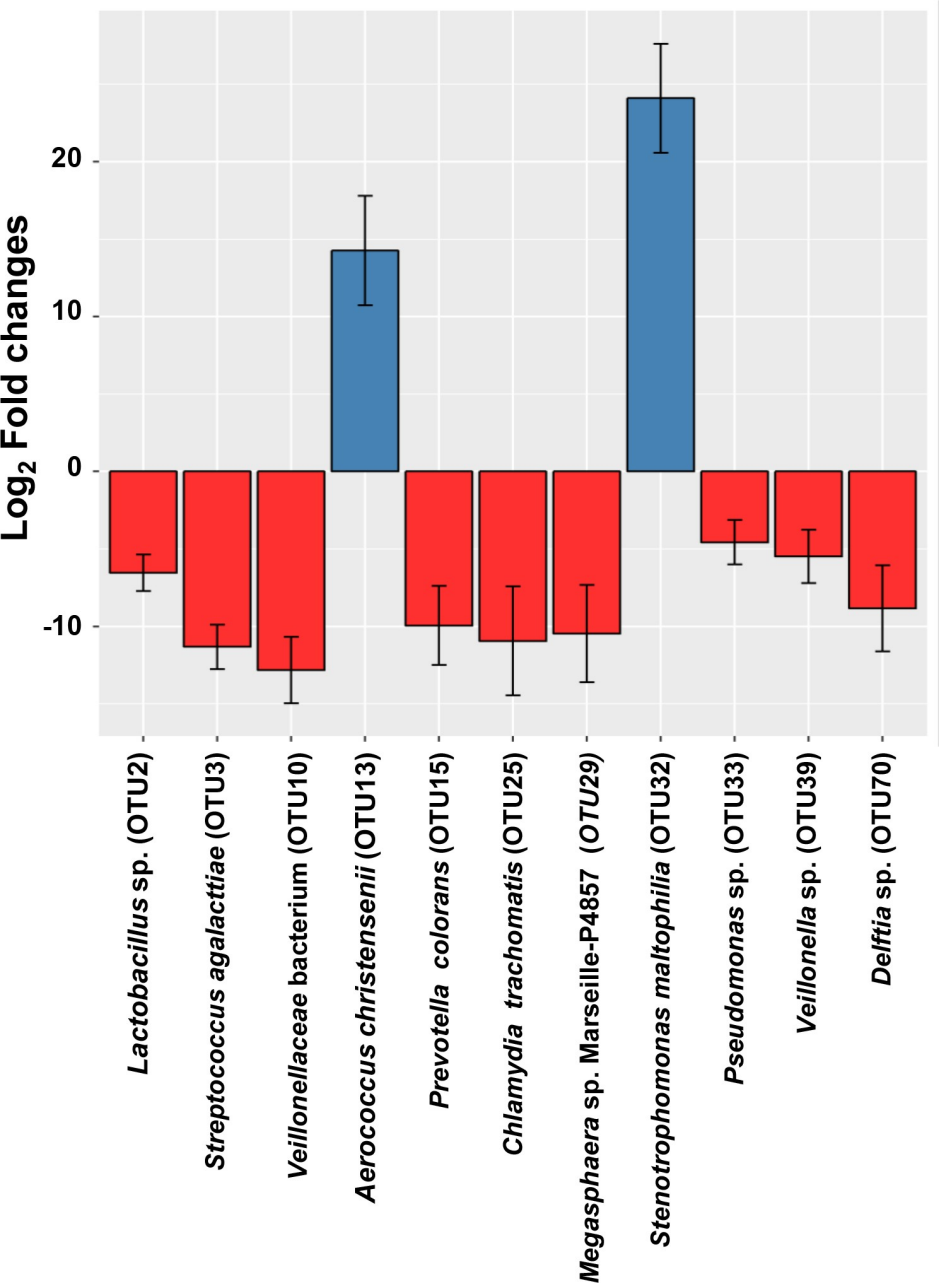
Significantly abundant taxa within the cervical groups of subjects with (+) and without (-) chlamydial infection. Analysis were done in all cohorts after accounting for differences in fertility status across the study groups. Red bars represent bacteria genus which were significantly abundant in *C*. *trachomatis*-infected group compared to non-chlamydial-infected group; whereas blue bars represent significant groups that are more abundant in non-chlamydial infected group. Numbers in bracket represents OTU.

After considering fertility status as a covariate on infection, we identified 11 differentially abundant taxa between *C*. *trachomatis*-positive and negative women (Fig 2). The composition of cervical microbiome for the subjects positive for *C*. *trachomatis* were associated with high taxa levels of a number of genera which included *Chlamydia* (OTU25), *Lactobacillus* (OTU2), *Streptococcus* (OTU3), *Megasphaera* (OTU10/29), *Prevotella_6* (OTU15), *Pseudomonas* (OTU33), *Veillonella* (OTU39), as well as *Delftia* (OTU70). Meanwhile, the genera *Aerococcus* (OTU13) and *Stenotrophomonas* (OTU32) were lower among subjects with *C*. *trachomatis* infection compared to those without chlamydial infection.

Sequence for each OTU was blasted using NCBI database to identify the microorganisms at species level. The species which matched the highest sequence homology with the input are as shown in Table 2. The abundant species in the *C*. *trachomatis* infected group were *Lactobacillus gallinarum or Lactobacillus crispatus* (OTU2), *Streptococcus agalactiae* (OTU3), *Prevotella colorans* (OTU15), *Pseudomonas mendocina* or *Psudomonas oleovorans* (OTU33), *Veillonella ratti* or *Veillonella seminalis* (OTU39), and *Delftia lacustris or Delftia tsuruhatensis* (OTU70). Whereas the abundant species in the non-chlamydial infected group were recognized as *Aerococcus christensenii* (OTU13), *Stenotrophomonas maltophilia* (OTU32). Some OTUs, for example OTU10 and OTU29, were unable to be identified at species level because of limited information in the database.

**Table 2:** BLAST results of differentially abundant operational taxonomic units (OTU). OTUs were searched against NCBI database using the BLASTn algorithm. The identified species with the highest homology and their accession numbers are listed. For BLAST results with more than one different species, only the top three species with the identical homology are displayed.

Operational Taxonomic Units (OTU)	Closest match	% Homology	*E*-value	Accession number
OTU2	*Lactobacillus gallinarum* strain PL53	464/465(99%)	0.0	MK182967.1
	*Lactobacillus crispatus* strain CO3MRSI	464/465(99%)	0.0	CP033426.1
	*Lactobacillus crispatus* strain HBUAS54364	464/465(99%)	0.0	MH819598.1
OTU3	*Streptococcus agalactiae* strain Israel1 16S ribosomal RNA gene, partial sequence	465/465(100%)	0.0	MK517599.1
	*Streptococcus agalactiae* strain TFJ0901 chromosome, complete genome	465/465(100%)	0.0	CP034315.1
	*Streptococcus agalactiae* strain FDAARGOS_512 chromosome, complete genome	465/465(100%)	0.0	CP033822.1
OTU10	*Veillonellaceae* bacterium DNF00751 16S ribosomal RNA gene, partial sequence	463/465(99%)	0.0	KF280299.1
	*Veillonellaceae* bacterium S3PF24 16S ribosomal RNA gene, partial sequence	463/465(99%)	0.0	JX104009.1
	*Veillonellaceae* bacterium S3PF10 16S ribosomal RNA gene, partial sequence	463/465(99%)	0.0	JX104005.1
OTU13	*Aerococcus christensenii* strain CCUG28831, complete genome	463/465(99%)	0.0	CP014159.1
	*Aerococcus christensenii* strain KA00635 16S ribosomal RNA gene, partial sequence	463/465(99%)	0.0	KP192302.1
	*Aerococcus* sp. CCUG28826 16S rRNA gene	463/465(99%)	0.0	Y17318.1
OTU15	*Prevotella colorans* strain A1336 16S ribosomal RNA, partial sequence	457/460(99%)	0.0	NR_151886.1
OTU25	*Chlamydia trachomatis* strain SQ19 chromosome, complete genome	464/465(99%)	0.0	CP017746.1
	*Chlamydia trachomatis* strain SQ28 genome	464/465(99%)	0.0	CP017744.1
	*Chlamydia trachomatis* strain SQ15 chromosome, complete genome	464/465(99%)	0.0	CP017745.1
OTU29	*Megasphaera* sp. Marseille-P4857 partial 16S rRNA gene, strain Marseille-P4857	465/465(100%)	0.0	LT960586.1
OTU32	*Stenotrophomonas maltophilia* strain MB33 16S ribosomal RNA gene, partial sequence	463/465(99%)	0.0	MH675502.1
	*Stenotrophomonas maltophilia* strain MB47 16S ribosomal RNA gene, partial sequence	463/465(99%)	0.0	MH196958.1
	*Stenotrophomonas maltophilia* strain MB42 16S ribosomal RNA gene, partial sequence	463/465(99%)	0.0	MH196954.1
OTU33	*Pseudomonas mendocina* strain C41 16S ribosomal RNA gene, partial sequence	463/465(99%)	0.0	MK182883.1
	*Pseudomonas oleovorans* subsp. oleovorans strain RS22 16S ribosomal RNA gene, partial sequence	463/465(99%)	0.0	MH715184.1
	*Pseudomonas mendocina* strain PDS_PXH_19PXH 16S ribosomal RNA gene, partial sequence	463/465(99%)	0.0	KY980683.1
OTU39	*Veillonella* sp. FFA-2014 strain 29/01/05-B-6143 16S ribosomal RNA gene, partial sequence	465/465(100%)	0.0	KJ580430.1
	*Veillonella ratti* strain JCM 6512 16S ribosomal RNA, partial sequence	465/465(100%)	0.0	NR_113377.1
	*Veillonella seminalis* strain ADV 4313.2 16S ribosomal RNA, partial sequence	465/465(100%)	0.0	NR_134226.1
OTU70	*Delftia lacustris* strain MB38 16S ribosomal RNA gene, partial sequence	463/465(99%)	0.0	MH675503.1
	*Delftia tsuruhatensis* strain IAE259 16S ribosomal RNA gene, partial sequence	463/465(99%)	0.0	MK414966.1
	*Delftia tsuruhatensis* strain ETEOC02 16S ribosomal RNA gene, partial sequence	463/465(99%)	0.0	MK590691.1

### Taxonomic diversity profiles of the endocervical microbiota in the presence of *C*. *trachomatis* infection

**Fig 3:**
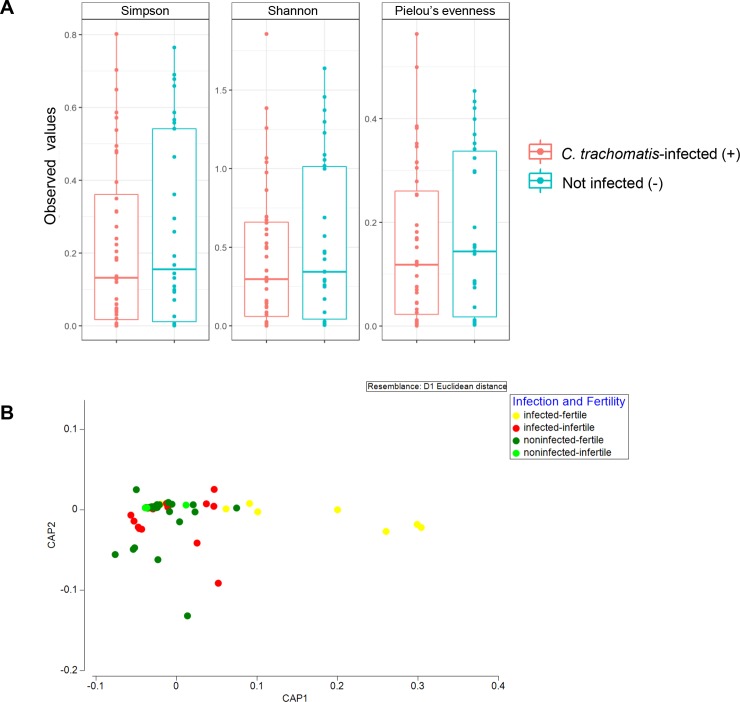
(A) Alpha diversity boxplots of the cervical microbiota community of subjects with and without diagnosis of *C*. *trachomatis*. Pielou’s evenness, Simpson’s, and Shannon’s indices were used to numerically measure species evenness and diversity across all sample groups. The alpha diversity for each group was not statistically different between groups. (B) Canonical analysis of principal coordinates (CAP) inferred based on log2 regularized OTUs data.

Alpha diversity measures including Pielou’s evenness alongside Simpson’s and Shannon’s indices were examined (Fig 3A). There is a general trend of higher alpha diversity indices in non-*C*. *trachomatis* infected than those in the *C*. *trachomatis*-infected subjects. However, the difference did not achieve statistical significance at alpha = 0.05 (Fig 3A). Beta diversity between the groups was further assessed by CAP (Fig 3B) and PERMANOVA analysis (Table 3). In CAP, separation between non-infected and infected groups was observed along axis CAP1. Indeed, a significance in bacterial compositional difference between groups with different infection status was apparent (pseudo-*F* = 1.4744, *P*_(perm)_ = 0.039). However, statistical significance was not achieved between groups with different fertility status (pseudo-*F* = 1.384, *P*(perm) = 0.079).

**Table 3:** Permutational multivariate analysis of variance (PERMANOVA) of the association between endocervical bacterial community composition and state of infection, as well fertility status.

Source	*df*	SS	MS	Pseudo-*F*	*P*_(perm)_	Unique perms
Infection status	1	4923.5	4923.5	1.4744	0.039*	998
Fertility status	1	4619.6	4619.6	1.3834	0.094	999
Residuals	67	2.23 × 10^5^	3339.4			
Total	69	2.31 × 10^5^				

*df* = degree of freedom; SS = sum of squares; MS = mean of squares; Pseudo-*F* = F value by permutation; *P*_(perm)_ = permutated *P*-value

*statistically significant (P < 0.05)

## Discussion

In this study, most of the patients diagnosed with *C*. *trachomatis* (88%) experienced primary or secondary infertility (100%, P<0.0001) as well as miscarriage (71%, P = 0.0089). A prior epidemiological study has indicated that women with genital *C*. *trachomatis* infection are 30% more likely to develop PID, ectopic pregnancy, as well as tubal factor infertility (TFI) than those without, whereas infections with *C*. *trachomatis* elevate the risk of PID by an additional 20% [33]. Our results are consistent with this finding, whereby a significant positive correlation between *C*. *trachomatis* and infertility was detected. Given the high prevalence of *C*. *trachomatis* infection and the severity of disease that ensues from the infection, a more effective screening and treatment strategy is warranted to curb the dissemination of *C*. *trachomatis* in the population. Notably, our study cohort comprised three major ethnic groups, namely the Malays, Chinese, Indians, as well as ethnic minority participants of other nationalities. However, due to the small sample size, the differences between ethnicities were not compared.

The vaginal flora, which constitutes 9% of the human microbiome, have been shown to play a critical role in the maintenance of overall health condition and disease state of the female reproductive tract [34]. Many studies have demonstrated that women with bacterial vaginosis are at higher risk of other sexually transmitted pathogens that includes *C*. *trachomatis*, signifying the importance of microbiota homeostasis and its role as a determinant in disease outcome [21, 23, 24, 35]. While the precise etiologic agents in bacterial vaginosis remain unclear, the prevailing view is that the disease is polymicrobial in nature caused by overgrowth of a consortium of anaerobic bacteria such as *Gardnerella vaginalis*, as well as microbes belonging to the genera *Streptococcus*, *Veillonella*, *Prevotella*, *Peptostreptococcus* and *Bacteroides* [36–38]. Although alpha-diversity analyses did not reveal categorical difference in cervical microbiota diversity across the sample groups, further evaluation of the cervical bacterial community using beta-diversity measure showed that there was significant difference in the taxonomic compositions between the subjects in relation to *C*. *trachomatis* infection (PERMANOVA, pseudo-*F* = 1.4744, *P*_(perm)_ = 0.039). In addition, we report here that women with genital chlamydial infection harbor significantly more abundant bacteria such as *Streptococcus agalactiae*, *Pseudomonas* sp., *Veillonellaceae* bacterium, *Megasphaera* sp., *Veillonella* sp., *Prevotella colorans*, *Veillonella* sp., as well as *Delftia* sp., the majority of these are anaerobes and facultative anaerobes which have been implicated in BV [36–39]. Other studies have also reported an increase in the colonization of diverse anaerobic bacteria in the cervicovaginal environment of women with *C*. *trachomatis* infection [26–28, 40, 41]. It is currently unknown whether *Delftia* can modulate the host susceptibility to other pathogens. *Delftia tsuruhatensis* appears to constitute the normal microbiota of the human vulva but there is evidence to suggest that its abundance in amniotic fluid and blood is associated with preterm birth [42, 43]. The significant presence of *Delftia* sp. in our *C*. *trachomatis*-positive group, therefore, merits further study to better elucidate its role in the female endocervical microbiota.

Differential abundance analysis (DESeq2) further showed that the taxon *Lactobacillus crispatus and L*. *gallinarum* was significantly elevated in the microbiota of women infected with *C*. *trachomatis* relative to those without *C*. *trachomatis* infection. Evidence is emerging that prevalence of particular *Lactobacillus* provides fewer protection compared to other members of the same genus. *L*. *crispatus* has been found to inhibit *C*. *trachomatis* infection, replication, as well as attachment to host cells; these effects are likely related to its production of lactic acid that lowers the surrounding pH and inhibits chlamydial growth [18–20]. However, it was reported recently that *L*. *crispatus* promotes repair of injured vaginal epithelial cells by promoting the secretion of vascular endothelial growth factor A (VEGF), which may help to reduce subsequent acquisition of urogenital tract infections [44]. *L*. *crispatus* was reported as one of the major compositions of endocervix microbiota among the asymptomatic *C*. *trachomatis*-infected patients in South Africa [28]. Meanwhile, *L*. *gallinarum* has previously been found in a high percentage of HPV-infected women in Beijing, China [45]. *L*. *iners* (OTU1) (data not shown) was the most abundant taxon in the endocervical milieu of both infected and non-infected group. Such dominance has been previously reported among normal healthy reproductive Thai and Chinese women [46, 47]. *L*. *iners* has been shown to be negatively associated with unfavorable reproductive health outcomes. Although commonly detected in the vagina of healthy reproductive age women [48], a high prevalence of *L*. *iners* has been shown to be a predictive factor for bacterial vaginosis and pre-term delivery [49–52]. In fact, *L*. *iners*-dominated microbiota has been linked to increased predisposition to acquisition of *C*. *trachomatis*, HIV, as well as *M*. *genitalium*, indicating its double-edged sword role in female reproductive health [26, 53–55]. However, we were unable to unequivocally determine the identity of the *Lactobacillus* in the present study as the sequences of *L*. *gallinarum* and *L*. *crispatus* had the identical homology in the BLAST results.

Conversely, subjects negative for *C*. *trachomatis* displayed significantly greater abundance of *Aerococcus christensenii*. Certain members of the genus *Aerococcus* including *A*. *urinae*, *A*. *sanguinocola*, and *A*. *viridans* are rare pathogens involved in urinary tract infections particularly among individuals with comorbidities [56–58]. In a similar study, Di Pietro et al. interrogated the microbial constituents of a population of Italian women infected with *C*. *trachomatis* and identified *A*. *christensenii* as the species closely associated with chlamydial infection [40]. Balle et al. showed that *A*. *christensenii* was only weakly related to *C*. *trachomatis* infection in a cohort of South African adolescents [28]. *A*. *christensenii* has previously been implicated as a cause of chorioamnionitis in a case report [59]. Beyond that, much remains unknown about the role of *A*. *christensenii* in the female genital tract. Currently, there is a paucity of information concerning the significance of *Stenotrophomonas* in the context of female reproductive health. *S*. *maltophilia* is considered as a human commensal and is a frequently isolated microorganism in urine specimens. Although rare, *S*. *maltophilia* has been reported to be a cause of nosocomial urinary tract infection [60, 61].

In conclusion, our study showed significant difference in endocervical microbiota between *C*. *trachomatis*-infected and non-infected cohorts among patients visiting gynecology clinics from Malaysia. However, we highlighted that genital *C*. *trachomatis* infection was not directly connected to increased cervical bacterial diversity, as no significant differences were observed in data richness and evenness. Further, we showed that infection with *C*. *trachomatis* was related to elevated prevalence of mostly strict and facultative anaerobes, however disruption of the sequencing data by PCR artefact should also be alerted [62]. Future studies involving a larger cohort with more stringent criteria will allow us to delineate the endocervical microbial landscape of women living in Malaysia. Nevertheless, the role of the anaerobes in disease progression is currently unknown, hence a further study to evaluate the involvement of taxa such as *A*. *christensenii* and *S*. *maltophilia* in chlamydial infection is warranted.

## Supporting information

S1 Table Primers used in the present study for diagnosis of *C*. *trachomatis*. (DOCX)

## References

[1] Newman L, Rowley J, Vander Hoorn S, Wijesooriya NS, Unemo M, Low N, et al Global Estimates of the Prevalence and Incidence of Four Curable Sexually Transmitted Infections in 2012 Based on Systematic Review and Global Reporting. PLoS One. 2015;10(12):e0143304 10.1371/journal.pone.0143304 26646541 PMC4672879

[2] Unemo M, Bradshaw CS, Hocking JS, de Vries HJC, Francis SC, Mabey D, et al Sexually transmitted infections: challenges ahead. Lancet Infect Dis. 2017;17(8):e235–e79. Epub 2017/07/14. 10.1016/S1473-3099(17)30310-9 . 28701272

[3] Ngeow YF, Rachagan SP, Ramachandran S. Prevalence of chlamydial antibody in Malaysians. J Clin Pathol. 1990;43(5):400–2. Epub 1990/05/01. 10.1136/jcp.43.5.400 2196283 PMC502444

[4] Ravindran J, Tan YI, Ngeow YF. The prevalence of *Chlamydia trachomatis* in patients with pelvic inflammatory disease. Med J Malaysia. 1998;53(1):16–21. Epub 2000/09/01. . 10968132

[5] Yeow TC, Wong WF, Sabet NS, Sulaiman S, Shahhosseini F, Tan GM, et al Prevalence of plasmid-bearing and plasmid-free *Chlamydia trachomatis* infection among women who visited obstetrics and gynecology clinics in Malaysia. BMC Microbiol. 2016;16:45 Epub 2016/03/19. 10.1186/s12866-016-0671-1 26987367 PMC4797335

[6] Lau CY, Qureshi AK. Azithromycin versus doxycycline for genital chlamydial infections: a meta-analysis of randomized clinical trials. Sex Transm Dis. 2002;29(9):497–502. Epub 2002/09/10. 10.1097/00007435-200209000-00001 . 12218839

[7] Miller KE. Diagnosis and treatment of *Chlamydia trachomatis* infection. Am Fam Physician. 2006;73(8):1411–6. . 16669564

[8] Spiliopoulou A, Lakiotis V, Vittoraki A, Zavou D, Mauri D. *Chlamydia trachomatis*: time for screening? Clin Microbiol Infect. 2005;11(9):687–9. 10.1111/j.1469-0691.2005.01187.x . 16104982

[9] Walker J, Tabrizi SN, Fairley CK, Chen MY, Bradshaw CS, Twin J, et al *Chlamydia trachomatis* incidence and re-infection among young women—behavioural and microbiological characteristics. PLoS One. 2012;7(5):e37778 10.1371/journal.pone.0037778 22662220 PMC3360595

[10] Brunham RC, Rey-Ladino J. Immunology of *Chlamydia* infection: implications for a *Chlamydia trachomatis* vaccine. Nat Rev Immunol. 2005;5(2):149–61. 10.1038/nri1551 . 15688042

[11] Moss NJ, Ahrens K, Kent CK, Klausner JD. The decline in clinical sequelae of genital *Chlamydia trachomatis* infection supports current control strategies. J Infect Dis. 2006;193(9):1336–8; author reply 8–9. 10.1086/503114 . 16586376

[12] Mackern-Oberti JP, Motrich RD, Breser ML, Sanchez LR, Cuffini C, Rivero VE. *Chlamydia trachomatis* infection of the male genital tract: an update. J Reprod Immunol. 2013;100(1):37–53. 10.1016/j.jri.2013.05.002 . 23870458

[13] Manavi K. A review on infection with *Chlamydia trachomatis*. Best Pract Res Clin Obstet Gynaecol. 2006;20(6):941–51. 10.1016/j.bpobgyn.2006.06.003 . 16934531

[14] Wilkowska-Trojniel M, Zdrodowska-Stefanow B, Ostaszewska-Puchalska I, Redzko S, Przepiesc J, Zdrodowski M. The influence of *Chlamydia trachomatis* infection on spontaneous abortions. Adv Med Sci. 2009;54(1):86–90. 10.2478/v10039-009-0008-5 . 19403438

[15] Rours GI, Duijts L, Moll HA, Arends LR, de Groot R, Jaddoe VW, et al *Chlamydia trachomatis* infection during pregnancy associated with preterm delivery: a population-based prospective cohort study. Eur J Epidemiol. 2011;26(6):493–502. 10.1007/s10654-011-9586-1 21538042 PMC3115062

[16] Kaminska D, Gajecka M. Is the role of human female reproductive tract microbiota underestimated? Benef Microbes. 2017;8(3):327–43. Epub 2017/05/16. 10.3920/BM2015.0174 . 28504576

[17] Cribby S, Taylor M, Reid G. Vaginal microbiota and the use of probiotics. Interdiscip Perspect Infect Dis. 2008;2008:256490 Epub 2008/01/01. 10.1155/2008/256490 19343185 PMC2662373

[18] Gong Z, Luna Y, Yu P, Fan H. Lactobacilli inactivate *Chlamydia trachomatis* through lactic acid but not H2O2. PLoS One. 2014;9(9):e107758 Epub 2014/09/13. 10.1371/journal.pone.0107758 25215504 PMC4162611

[19] Mastromarino P, Di Pietro M, Schiavoni G, Nardis C, Gentile M, Sessa R. Effects of vaginal lactobacilli in *Chlamydia trachomatis* infection. Int J Med Microbiol. 2014;304(5–6):654–61. Epub 2014/05/31. 10.1016/j.ijmm.2014.04.006 . 24875405

[20] Nardini P, Nahui Palomino RA, Parolin C, Laghi L, Foschi C, Cevenini R, et al *Lactobacillus crispatus* inhibits the infectivity of *Chlamydia trachomatis* elementary bodies, in vitro study. Sci Rep. 2016;6:29024 Epub 2016/06/30. 10.1038/srep29024 27354249 PMC4926251

[21] Gillet E, Meys JF, Verstraelen H, Bosire C, De Sutter P, Temmerman M, et al Bacterial vaginosis is associated with uterine cervical human papillomavirus infection: a meta-analysis. BMC Infect Dis. 2011;11:10 Epub 2011/01/13. 10.1186/1471-2334-11-10 21223574 PMC3023697

[22] Brotman RM, Bradford LL, Conrad M, Gajer P, Ault K, Peralta L, et al Association between *Trichomonas vaginalis* and vaginal bacterial community composition among reproductive-age women. Sex Transm Dis. 2012;39(10):807–12. Epub 2012/09/26. 10.1097/OLQ.0b013e3182631c79 23007708 PMC3458234

[23] Gallo MF, Macaluso M, Warner L, Fleenor ME, Hook EW 3rd, Brill I, et al Bacterial vaginosis, gonorrhea, and chlamydial infection among women attending a sexually transmitted disease clinic: a longitudinal analysis of possible causal links. Ann Epidemiol. 2012;22(3):213–20. Epub 2011/12/24. 10.1016/j.annepidem.2011.11.005 . 22192490

[24] Bautista CT, Wurapa EK, Sateren WB, Morris SM, Hollingsworth BP, Sanchez JL. Association of Bacterial Vaginosis With *Chlamydia* and Gonorrhea Among Women in the U.S. Army. Am J Prev Med. 2017;52(5):632–9. Epub 2016/11/07. 10.1016/j.amepre.2016.09.016 . 27816380

[25] Gosmann C, Anahtar MN, Handley SA, Farcasanu M, Abu-Ali G, Bowman BA, et al Lactobacillus-Deficient Cervicovaginal Bacterial Communities Are Associated with Increased HIV Acquisition in Young South African Women. Immunity. 2017;46(1):29–37. 10.1016/j.immuni.2016.12.013 28087240 PMC5270628

[26] van der Veer C, Bruisten SM, van der Helm JJ, de Vries HJ, van Houdt R. The Cervicovaginal Microbiota in Women Notified for *Chlamydia trachomatis* Infection: A Case-Control Study at the Sexually Transmitted Infection Outpatient Clinic in Amsterdam, The Netherlands. Clin Infect Dis. 2017;64(1):24–31. Epub 2016/08/28. 10.1093/cid/ciw586 . 27567124

[27] Filardo S, Di Pietro M, Porpora MG, Recine N, Farcomeni A, Latino MA, et al Diversity of Cervical Microbiota in Asymptomatic *Chlamydia trachomatis* Genital Infection: A Pilot Study. Front Cell Infect Microbiol. 2017;7:321 Epub 2017/08/05. 10.3389/fcimb.2017.00321 28770172 PMC5509768

[28] Balle C, Lennard K, Dabee S, Barnabas SL, Jaumdally SZ, Gasper MA, et al Endocervical and vaginal microbiota in South African adolescents with asymptomatic *Chlamydia trachomatis* infection. Sci Rep. 2018;8(1):11109 Epub 2018/07/25. 10.1038/s41598-018-29320-x 30038262 PMC6056523

[29] Tamarelle J, de Barbeyrac B, Le Hen I, Thiebaut A, Bebear C, Ravel J, et al Vaginal microbiota composition and association with prevalent *Chlamydia trachomatis* infection: a cross-sectional study of young women attending a STI clinic in France. Sex Transm Infect. 2018 Epub 2018/01/24. 10.1136/sextrans-2017-053346 . 29358524

[30] Schloss PD, Westcott SL, Ryabin T, Hall JR, Hartmann M, Hollister EB, et al Introducing mothur: open-source, platform-independent, community-supported software for describing and comparing microbial communities. Appl Environ Microbiol. 2009;75(23):7537–41. Epub 2009/10/06. 10.1128/AEM.01541-09 PubMed Central PMCID: PMC2786419. 19801464 PMC2786419

[31] McMurdie PJ, Holmes S. phyloseq: an R package for reproducible interactive analysis and graphics of microbiome census data. PLoS One. 2013;8(4):e61217 Epub 2013/05/01. 10.1371/journal.pone.0061217 23630581 PMC3632530

[32] Love MI, Huber W, Anders S. Moderated estimation of fold change and dispersion for RNA-seq data with DESeq2. Genome Biol. 2014;15(12):550 Epub 2014/12/18. 10.1186/s13059-014-0550-8 25516281 PMC4302049

[33] Davies B, Turner KM, Frolund M, Ward H, May MT, Rasmussen S, et al Risk of reproductive complications following chlamydia testing: a population-based retrospective cohort study in Denmark. Lancet Infect Dis. 2016;16(9):1057–64. 10.1016/S1473-3099(16)30092-5 . 27289389

[34] Group NHW, Peterson J, Garges S, Giovanni M, McInnes P, Wang L, et al The NIH Human Microbiome Project. Genome Res. 2009;19(12):2317–23. Epub 2009/10/13. 10.1101/gr.096651.109 19819907 PMC2792171

[35] Wiesenfeld HC, Hillier SL, Krohn MA, Landers DV, Sweet RL. Bacterial vaginosis is a strong predictor of *Neisseria gonorrhoeae* and *Chlamydia trachomatis* infection. Clin Infect Dis. 2003;36(5):663–8. Epub 2003/02/21. 10.1086/367658 . 12594649

[36] Biagi E, Vitali B, Pugliese C, Candela M, Donders GG, Brigidi P. Quantitative variations in the vaginal bacterial population associated with asymptomatic infections: a real-time polymerase chain reaction study. Eur J Clin Microbiol Infect Dis. 2009;28(3):281–5. Epub 2008/09/03. 10.1007/s10096-008-0617-0 . 18762999

[37] Turovskiy Y, Sutyak Noll K, Chikindas ML. The aetiology of bacterial vaginosis. J Appl Microbiol. 2011;110(5):1105–28. Epub 2011/02/22. 10.1111/j.1365-2672.2011.04977.x 21332897 PMC3072448

[38] Nelson TM, Borgogna JL, Brotman RM, Ravel J, Walk ST, Yeoman CJ. Vaginal biogenic amines: biomarkers of bacterial vaginosis or precursors to vaginal dysbiosis? Front Physiol. 2015;6:253 Epub 2015/10/21. 10.3389/fphys.2015.00253 26483694 PMC4586437

[39] Ranjit E, Raghubanshi BR, Maskey S, Parajuli P. Prevalence of Bacterial Vaginosis and Its Association with Risk Factors among Nonpregnant Women: A Hospital Based Study. Int J Microbiol. 2018;2018:8349601 Epub 2018/04/26. 10.1155/2018/8349601 29692813 PMC5859802

[40] Di Pietro M, Filardo S, Porpora MG, Recine N, Latino MA, Sessa R. HPV/*Chlamydia trachomatis* co-infection: metagenomic analysis of cervical microbiota in asymptomatic women. New Microbiol. 2018;41(1):34–41. Epub 2018/01/10. . 29313867

[41] Witkin SS. Vaginal microbiome studies in pregnancy must also analyse host factors. BJOG. 2019;126(3):359 Epub 2018/05/24. 10.1111/1471-0528.15300 . 29791773

[42] DiGiulio DB, Romero R, Amogan HP, Kusanovic JP, Bik EM, Gotsch F, et al Microbial prevalence, diversity and abundance in amniotic fluid during preterm labor: a molecular and culture-based investigation. PLoS One. 2008;3(8):e3056 Epub 2008/08/30. 10.1371/journal.pone.0003056 18725970 PMC2516597

[43] You Y-A, Yoo JY, Kwon EJ, Kim YJ. Blood Microbial Communities During Pregnancy Are Associated With Preterm Birth. Frontiers in Microbiology. 2019;10(1122). 10.3389/fmicb.2019.01122 31214131 PMC6558066

[44] Takada K, Komine-Aizawa S, Kuramochi T, Ito S, Trinh QD, Pham NTK, et al *Lactobacillus crispatus* accelerates re-epithelialization in vaginal epithelial cell line MS74. Am J Reprod Immunol. 2018;80(3):e13027 Epub 2018/08/26. 10.1111/aji.13027 . 30144195

[45] Gao W, Weng J, Gao Y, Chen X. Comparison of the vaginal microbiota diversity of women with and without human papillomavirus infection: a cross-sectional study. BMC Infect Dis. 2013;13:271 Epub 2013/06/14. 10.1186/1471-2334-13-271 23758857 PMC3684509

[46] Shi Y, Chen L, Tong J, Xu C. Preliminary characterization of vaginal microbiota in healthy Chinese women using cultivation-independent methods. J Obstet Gynaecol Res. 2009;35(3):525–32. Epub 2009/06/17. 10.1111/j.1447-0756.2008.00971.x . 19527394

[47] Sirichoat A, Buppasiri P, Engchanil C, Namwat W, Faksri K, Sankuntaw N, et al Characterization of vaginal microbiota in Thai women. PeerJ. 2018;6:e5977 Epub 2018/12/01. 10.7717/peerj.5977 30498641 PMC6252066

[48] Ravel J, Gajer P, Abdo Z, Schneider GM, Koenig SS, McCulle SL, et al Vaginal microbiome of reproductive-age women. Proc Natl Acad Sci U S A. 2011;108 Suppl 1:4680–7. Epub 2010/06/11. 10.1073/pnas.1002611107 20534435 PMC3063603

[49] Tamrakar R, Yamada T, Furuta I, Cho K, Morikawa M, Yamada H, et al Association between *Lactobacillus* species and bacterial vaginosis-related bacteria, and bacterial vaginosis scores in pregnant Japanese women. BMC Infect Dis. 2007;7:128 Epub 2007/11/08. 10.1186/1471-2334-7-128 17986357 PMC2212641

[50] Macklaim JM, Gloor GB, Anukam KC, Cribby S, Reid G. At the crossroads of vaginal health and disease, the genome sequence of *Lactobacillus iners* AB-1. Proc Natl Acad Sci U S A. 2011;108 Suppl 1:4688–95. Epub 2010/11/10. 10.1073/pnas.1000086107 21059957 PMC3063587

[51] Kindinger LM, Bennett PR, Lee YS, Marchesi JR, Smith A, Cacciatore S, et al The interaction between vaginal microbiota, cervical length, and vaginal progesterone treatment for preterm birth risk. Microbiome. 2017;5(1):6 Epub 2017/01/21. 10.1186/s40168-016-0223-9 28103952 PMC5244550

[52] Stafford GP, Parker JL, Amabebe E, Kistler J, Reynolds S, Stern V, et al Spontaneous Preterm Birth Is Associated with Differential Expression of Vaginal Metabolites by *Lactobacilli*-Dominated Microflora. Front Physiol. 2017;8:615 Epub 2017/09/08. 10.3389/fphys.2017.00615 28878691 PMC5572350

[53] Borgdorff H, Tsivtsivadze E, Verhelst R, Marzorati M, Jurriaans S, Ndayisaba GF, et al *Lactobacillus*-dominated cervicovaginal microbiota associated with reduced HIV/STI prevalence and genital HIV viral load in African women. ISME J. 2014;8(9):1781–93. Epub 2014/03/07. 10.1038/ismej.2014.26 24599071 PMC4139719

[54] Molenaar MC, Singer M, Ouburg S. The two-sided role of the vaginal microbiome in *Chlamydia trachomatis* and *Mycoplasma genitalium* pathogenesis. J Reprod Immunol. 2018;130:11–7. Epub 2018/08/28. 10.1016/j.jri.2018.08.006 . 30149363

[55] van Houdt R, Ma B, Bruisten SM, Speksnijder A, Ravel J, de Vries HJC. *Lactobacillus iners*-dominated vaginal microbiota is associated with increased susceptibility to *Chlamydia trachomatis* infection in Dutch women: a case-control study. Sex Transm Infect. 2018;94(2):117–23. Epub 2017/09/28. 10.1136/sextrans-2017-053133 28947665 PMC6083440

[56] Mohan B, Zaman K, Anand N, Taneja N. *Aerococcus Viridans*: A Rare Pathogen Causing Urinary Tract Infection. J Clin Diagn Res. 2017;11(1):DR01–DR3. Epub 2017/03/10. 10.7860/JCDR/2017/23997.9229 28273968 PMC5324413

[57] Higgins A, Garg T. *Aerococcus urinae*: An Emerging Cause of Urinary Tract Infection in Older Adults with Multimorbidity and Urologic Cancer. Urol Case Rep. 2017;13:24–5. Epub 2017/04/25. 10.1016/j.eucr.2017.03.022 28435789 PMC5393163

[58] Zhang Q, Kwoh C, Attorri S, Clarridge JE 3rd. *Aerococcus urinae* in urinary tract infections. J Clin Microbiol. 2000;38(4):1703–5. Epub 2000/04/04. 10747177 10.1128/jcm.38.4.1703-1705.2000 PMC86536

[59] Carlstein C, Marie Soes L, Jorgen Christensen J. *Aerococcus christensenii* as Part of Severe Polymicrobial Chorioamnionitis in a Pregnant Woman. Open Microbiol J. 2016;10:27–31. Epub 2016/03/26. 10.2174/1874285801610010027 27014376 PMC4787314

[60] Vartivarian SE, Papadakis KA, Anaissie EJ. *Stenotrophomonas (Xanthomonas) maltophilia* urinary tract infection. A disease that is usually severe and complicated. Arch Intern Med. 1996;156(4):433–5. Epub 1996/02/26. . 8607729

[61] Denton M, Kerr KG. Microbiological and clinical aspects of infection associated with *Stenotrophomonas maltophilia*. Clin Microbiol Rev. 1998;11(1):57–80. Epub 1998/02/11. 9457429 10.1128/cmr.11.1.57 PMC121376

[62] Kebschull JM, Zador AM. Sources of PCR-induced distortions in high-throughput sequencing data sets. Nucleic Acids Res. 2015;43(21):e143 Epub 2015/07/19. 10.1093/nar/gkv717 26187991 PMC4666380

