# Supplementary material for: Diversity of endocervical microbiota associated with genital Chlamydia trachomatis infection and infertility among women visiting obstetrics and gynecology clinics in Malaysia
Source: PLoS One. 2019 Nov 18;14(11):e0224658. doi: 10.1371/journal.pone.0224658 (PMC6860443; doi:10.1371/journal.pone.0224658)
Supplement: S1 Table — (DOCX) [file pone.0224658.s001.docx]

**Supporting Information**

**S1 Table.** Primers used in the present study for diagnosis of *C. trachomatis*.

| **Target gene** | **Primer** | **Sequence (5’-3’)** | **Amplicon size** |
| --- | --- | --- | --- |
| *MOMP* | Outer forward | TTGTTTTCGACCGTGTTTTG | 455 bp |
|  | Outer reverse | AGCRTATTGGAAAGAAGCBCCTAA |  |
|  | Inner forward | AAACWGATGTGAATAAAGARTT | 395 bp |
|  | Inner reverse | TCCCASARAGCTGCDCGAGC |  |
| *Pgp8* | Outer forward | TTGGCYGCTAGAAAAGGCGATT | 212 bp |
|  | Outer reverse | TCCGGAACAYATGATGCGAAGT |  |
|  | Inner forward | AACCAAGGTCGATGTGATAG | 150 bp |
|  | Inner reverse | TCAGATAATTGGCGATTCTT |  |
| *Pgp1* | Forward | TTCTTTGATGGCTTCCCAAC | 456 bp |
|  | Reverse | ACGATTTTCTCCAACCGATG |  |
| β-globin | Forward | GAAGAGCCAAGGACAGGTAC | 268 bp |
|  | Reverse | CAACTTCATCCACGTTCACC |  |
